# Combined [68 Ga]Ga-PSMA-11 and low-dose 2-[18F]FDG PET/CT using a long-axial field of view scanner for patients referred for [177Lu]-PSMA-radioligand therapy

**DOI:** 10.1007/s00259-022-05961-z

**Published:** 2022-09-22

**Authors:** Ian Alberts, Robin Schepers, Konstantinos Zeimpekis, Hasan Sari, Axel Rominger, Ali Afshar-Oromieh

**Affiliations:** 1 Department of Nuclear Medicine, Inselspital, Bern University Hospital, University of Bern, Freiburgstr. 18, 3010 Bern, Switzerland; 2 Advanced Clinical Imaging Technology, Siemens Healthcare AG, Lausanne, Switzerland

**Keywords:** Total-body PET, Ultra-long FOV PET, Whole-body PET, PET/CT, Positron-emission-tomography, Digital PET

## Abstract

**Purpose:**

Performing 2-[^18^F]FDG PET/CT in addition to a PSMA-ligand PET/CT can assist in the detection of lesions with low PSMA expression and may help in prognostication and identification of patients who likely benefit from PSMA-radioligand therapy (PSMA-RLT). However, the cost and time needed for a separate PET/CT examination might hinder its routine implementation. In this communication, we present our initial experiences with additional low-dose 2-[^18^F]FDG PET/CT as part of a dual-tracer and same-day imaging protocol which exploits the higher sensitivity exhibited by long-axial field-of-view (LAFOV) and total-body PET/CT systems and demonstrates its feasibility.

**Methods:**

Fourteen patients referred for evaluation for PSMA-RLT received [^68^ Ga]Ga-PSMA-11 PET/CT at 1 h p.i. with a standard activity of 150 MBq and an additional low-dose 2-[^18^F]FDG PET/CT with 40 MBq 1 h thereafter using a long-axial field-of-view PET/CT system in a single sitting and as per institutional protocol. Scans were scrutinized by two experienced nuclear medicine physicians for mismatch findings.

**Results:**

The combined protocol identified additional lesions with low or absent PSMA-expression but high FDG-avidity in 1/14 (7%) patients. The protocol was easily implemented and well tolerated by all patients.

**Conclusion:**

Additional low-dose 2-[^18^F]FDG-PET/CT is feasible as part of a same-day imaging protocol and can help reveal lesions of low PSMA avidity as part of therapy assessment for [^177^Lu]-PSMA radioligand therapy and demonstrates higher sensitivity compared to [^68^ Ga]Ga-PSMA-11 PET/CT alone in some patients.

## Introduction

[^177^Lu]-labelled PSMA-radioligand therapy (PSMA-RLT) represents a life-prolonging treatment for men with metastatic castration resistance prostate cancer (mCRPC) [1] and is now under investigation for its possible role in metastatic hormone sensitive prostate cancer (mHSPC) [2]. Despite the high efficacy and low toxicity of [^177^Lu]-PSMA-RLT [3], selection of patients who stand to benefit from therapy remains challenging. A recent analysis of screen failures from the VISION trial demonstrated poorer outcomes for those classified as ineligible based on baseline PSMA-PET imaging [4], highlighting the importance of adequate theragnostic assessment of patients prior to PSMA-RLT in patient selection and prognostication; patients who exhibit lesions with low PSMA-expression are unlikely to clinically benefit from the procedure.

However, what constitutes adequate or optimal assessment is controversial. Whereas the phase II trial published by Hofman et al. included additional 2-[^18^F]FDG-PET/CT (FDG-PET/CT) to identify lesions with low PSMA-expression [3], extant EANM procedural guidelines suggest a number of viable imaging modalities to identify non-PSMA avid sites of active disease, including bone scan, MRI and contrast-enhanced CT (ceCT) as well as FDG-PET/CT [5]. Not all centres routinely perform additional FDG-PET/CT, and reimbursement of an additional PET imaging modality is challenging in many jurisdictions [6]. At present, patients referred for PSMA-RLT have exhausted multiple therapeutic options and are approaching the end-stage of their disease. In this context, the need for a separate imaging visit requiring a prolonged fast and journey to a specialist nuclear medicine imaging center represents an additional and potentially unwelcome burden for these patients.

Recently introduced long-axial field-of-view (LAFOV) PET/CT scanners demonstrate substantially higher sensitivity compared to previous generation analogue and short-axial FOV systems [7–11] and which we have previously shown to be useful for [^68^ Ga]Ga-PSMA-11 PET/CT at low activities with high diagnostic quality [12]. This higher sensitivity can be exploited to afford an additional FDG-PET/CT with low applied activities (with low equivalent dose, herein referred to as “low-dose”) and as part of the same imaging session. The aim of this short communication is to propose an imaging protocol which combines PSMA and FDG-PET/CT in a single imaging session.

## Materials and methods

### Study design

This retrospective short communication presents 14 cases who underwent standard clinical [^68^ Ga]Ga-PSMA-11 PET/CT on our LAFOV system between January and December 2021, where imaging was performed for therapeutic assessment and where additional imaging was performed with a low-dose FDG-PET/CT in the same imaging session.

### Imaging procedures

All scans were performed using the Siemens Biograph Vision Quadra system (axial FOV = 106 cm). Reconstruction parameters were as previously described [12]. Patients first received a PET/CT with [^68^ Ga]Ga-PSMA-11 using a standard activity (150 ± 15% MBq) at 1 h p.i. with a scan time of 5 min at 1 h [13] and in a single bed position of 106 cm (vertex to thighs). The radiopharmaceutical was prepared as previously published [14]. Immediately upon completion of this scan, 40 MBq of 2-[^18^F]FDG was administered and a second PET/CT performed 1 h thereafter with a 15-min scan time in a single bed position of 106 cm (vertex to thighs). Reconstruction parameters were as previously described [7, 15]. The total time for the patient which was required for the imaging session therefore took approximately 2 h 15 min. Both scans were performed as clinical routine and patients arrived prepared for the 2-[^18^F]FDG examination in a fasted state (> 4 h), no administration of insulin in the previous 4 h and with confirmation of a blood sugar < 11.0 mmol/l in accordance with EANM guidelines [16].

### Analysis

Both scans were analysed by two qualified nuclear medicine physicians (first and last authors) and mismatch lesions (FDG-avid but with PSMA uptake below the level of the liver) were noted. Semi-quantitative analysis of the images was precluded by the superimposition of the two tracers at the second scan.

## Results

The protocol was implemented in 14 patients undergoing PSMA-RLT at our center during the above mentioned time window. All patients tolerated the protocol well and were compliant with instructions for pre-scan fasting. A physiological distribution was noted for the additional low-dose FDG-PET/CT, with FDG-avid and non-PSMA-accumulating organs such as the brain showing adequate FDG uptake. Subjective image quality was noted to be excellent for both scans.

In 13 patients, no mismatch findings could be identified. In *n* = 1 patient (7%), additional lesions were identified with either absent or low PSMA uptake but high FDG avidity. The following clinical vignettes will demonstrate the feasibility and value of the protocol by means of an example patient with mismatch findings and a patient with no mismatch findings.

### Clinical vignette 1—example patient with mismatch findings

**Fig. 1:**
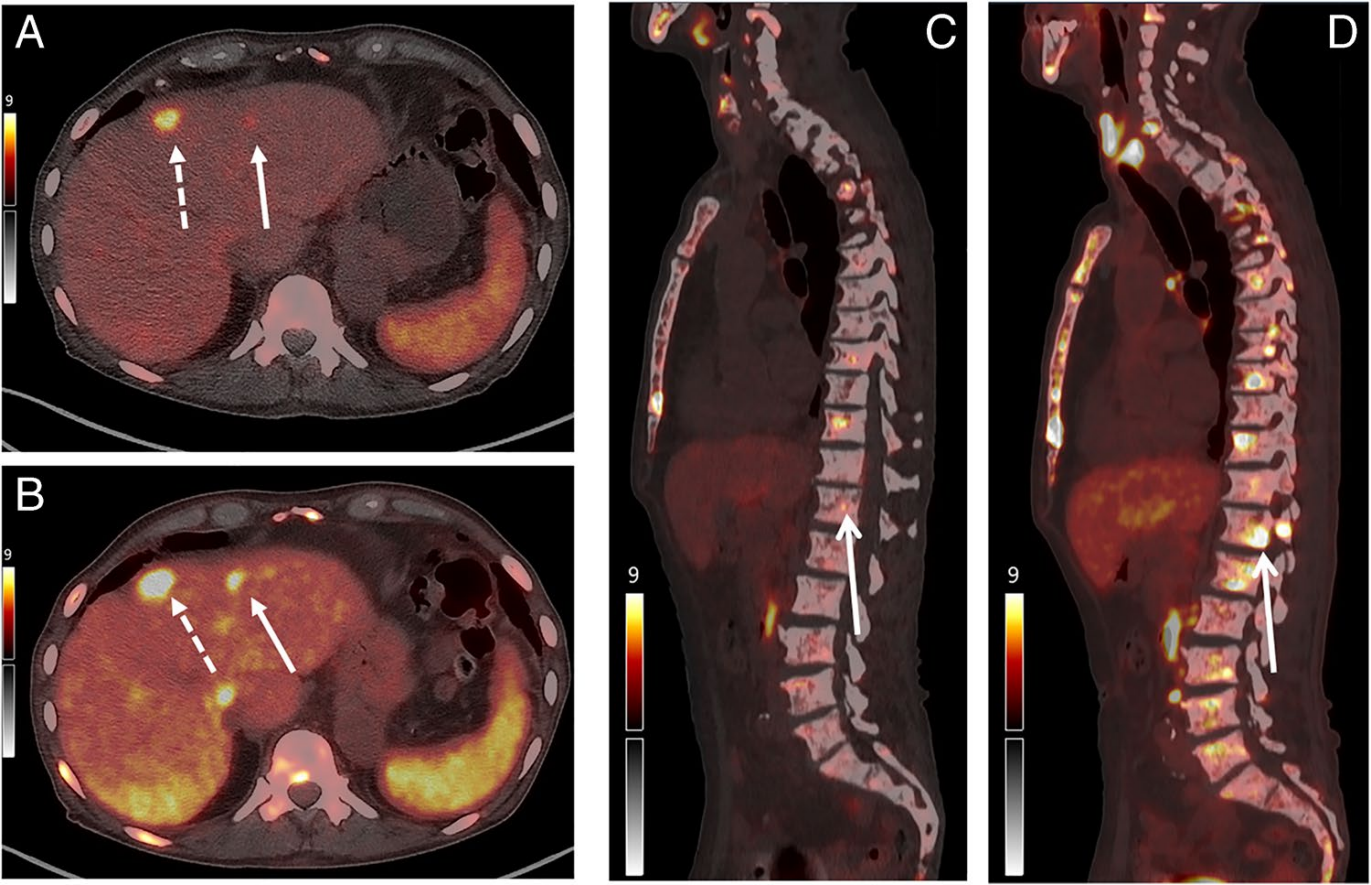
Example images for a patient with mismatch lesions. **A** and **C** show the [^68^ Ga]Ga-PSMA-11 PET/CT performed at 1 h p.i. and **B** and **D** the additional PET/CT 1 h following additional administration of FDG. Although the lesion shown by the broken arrow demonstrates increased uptake at second imaging, the hepatic lesion shown by the solid white arrow in **A** and the example bone lesion in **C** show intense FDG uptake at the second PET/CT (**B** and **D**) which is indicative of low PSMA expression and high FDG avidity

**Fig. 2:**
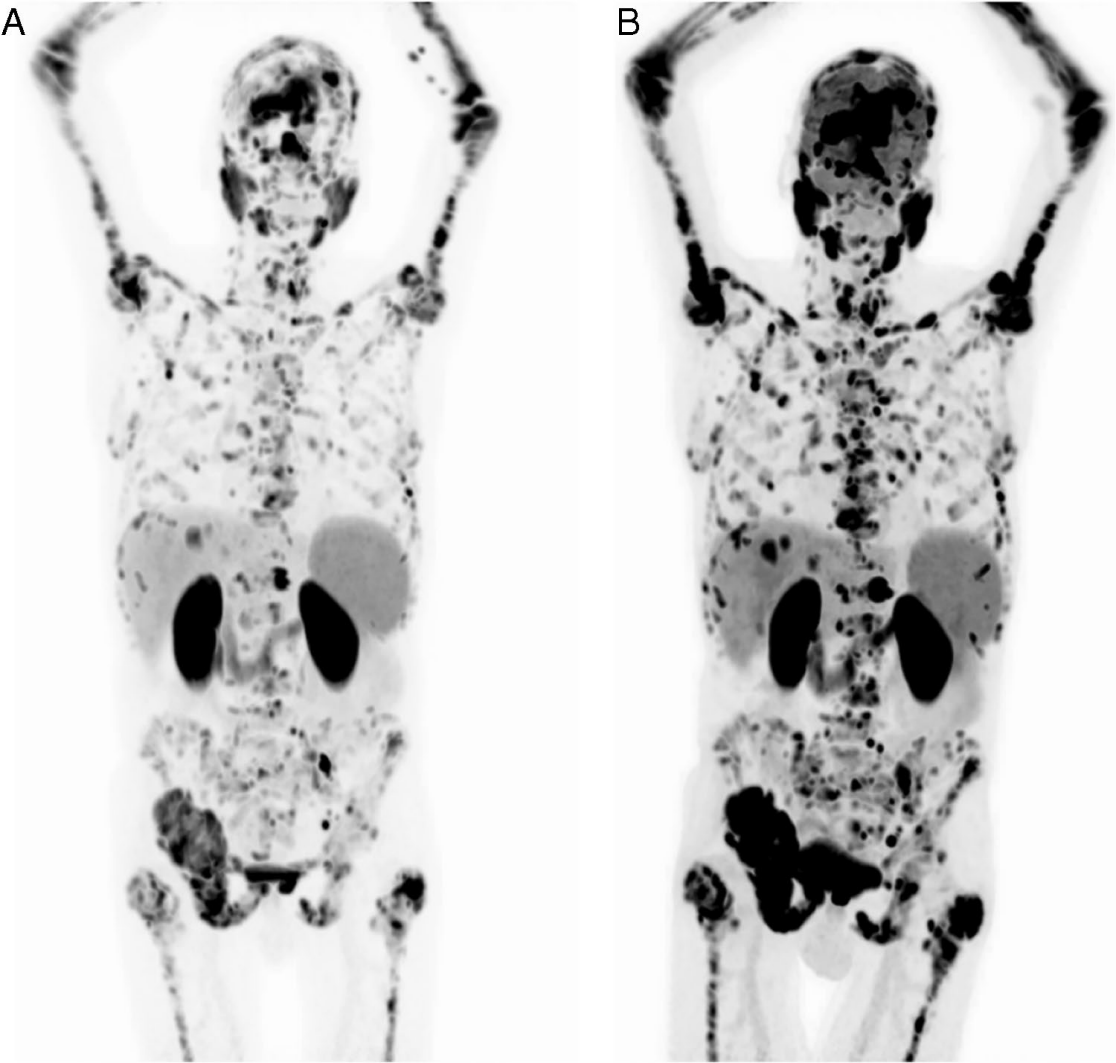
MIP images for **A** [^68^ Ga]Ga-PSMA-11 PET/CT and **B** FDG of the patient included in Fig. 1

This 76-year-old patient was referred for evaluation for PSMA-RLT for mCRPC with known bone and liver metastases. [^68^ Ga]Ga-PSMA-11 PET/CT showed multiple bone metastases with heterogeneous and low to intense PSMA-expression (SUVmax range 3.2–9.2) and heterogeneous liver metastases, some of which exhibited tracer uptake only partially above the liver background (SUVmax healthy liver 2.9, SUVmax lesion in left liver lobe 4.9). In the following additional FDG-PET/CT, intense uptake is now seen in the areas of low avidity in the [68 Ga]Ga-PSMA-11 PET/CT and is demonstrated in the example images in Fig. 1 and the maximum intensity projections (MIP) in Fig. 2. These findings are consistent with lesions with reduced PSMA expression and high FDG avidity. In interdisciplinary discussion with the oncologist and discussion with the patient, it was decided not to perform PSMA-RLT for this patient because of the low PSMA avidity of the multiple osseous metastases.

### Clinical vignette 2—example patient with no mismatch findings

**Fig. 3:**
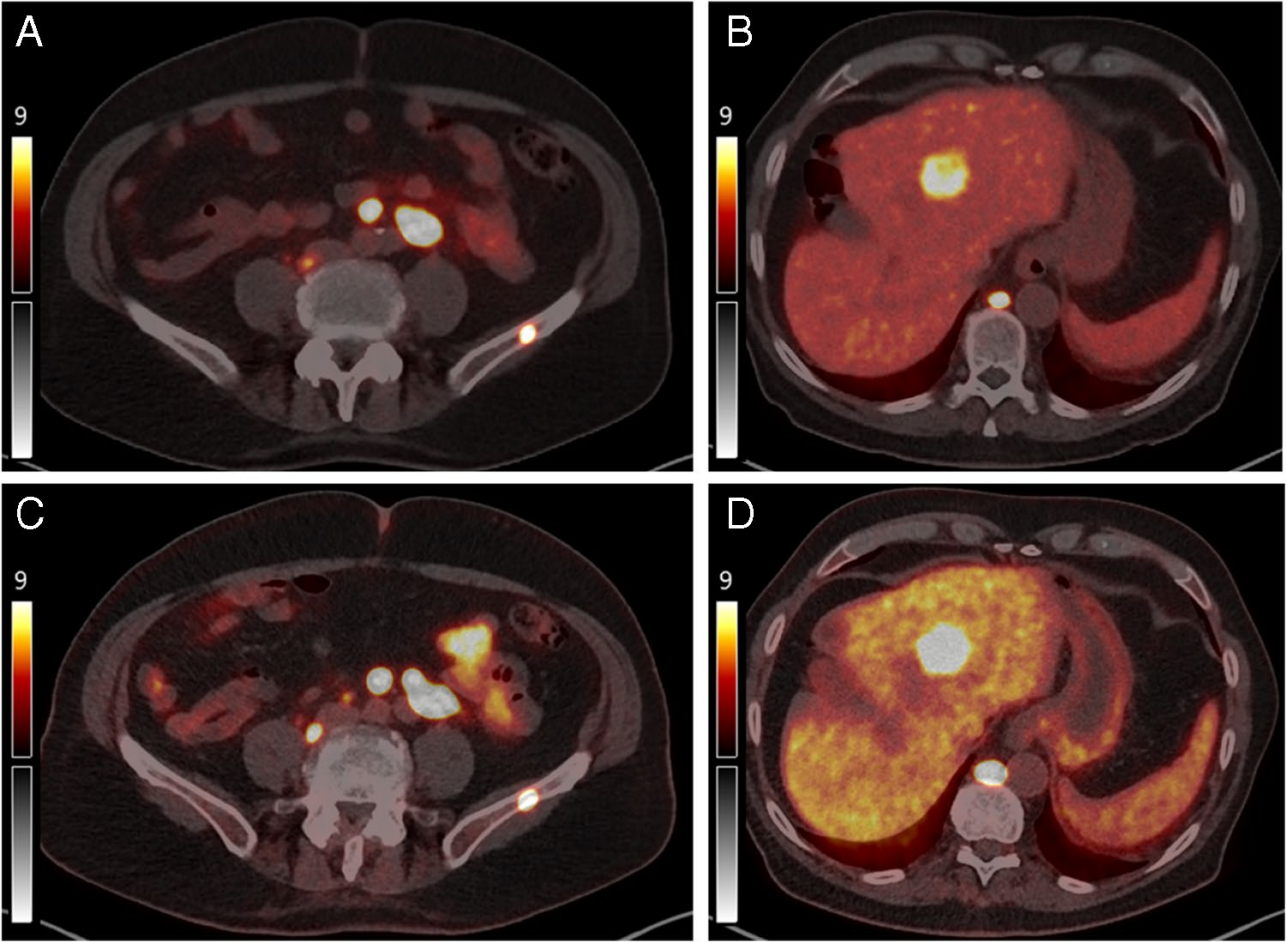
Example images for patient without mismatch lesions. **A** and **B** show images post-[^68^ Ga]Ga-PSMA-11 PET/T and **C** and **D** post-additional PET/CT with FDG. All lesions (lymph nodes, bone, and liver) demonstrate intense uptake in both scans. No new lesions are visible at additional imaging with FDG

**Fig. 4:**
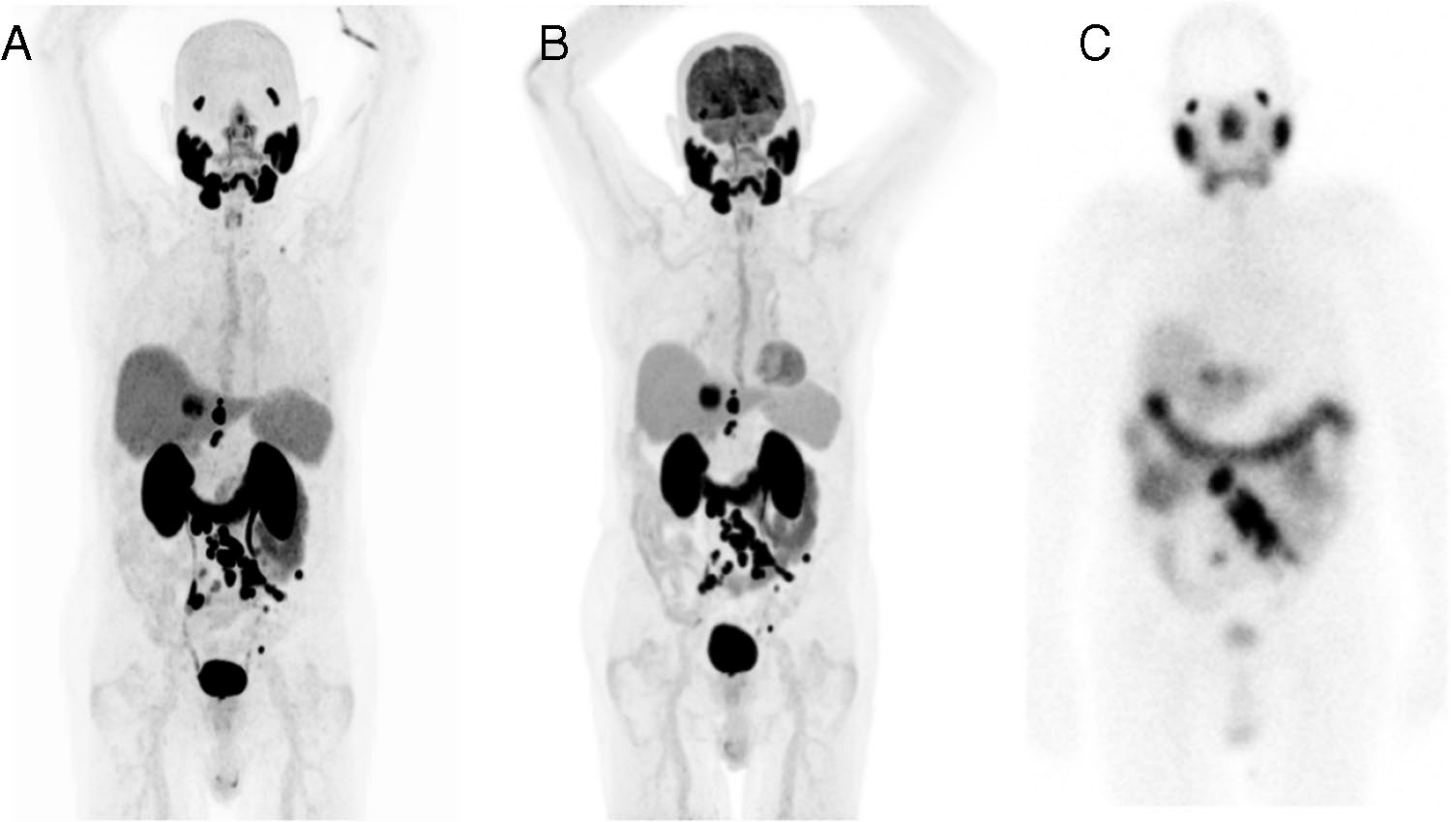
Example MIPs of the patient mentioned in Fig. 3 for **A** [^68^ Ga]Ga-PSMA-11 and **B** FDG showing no mismatch lesions. All lesions demonstrated good uptake of [^177^Lu]Lu-PSMA-617 in the post-therapy scintigraphy (**C**)

This 81-year-old patient was also referred for evaluation of PSMA-RLT for mCRPC. He had received palliative radiotherapy two years previously and was currently receiving leuprolin and abiraterone. The example images from the [^68^ Ga]Ga-PSMA-11 PET/CT and FDG-PET/CT shown in Fig. 3 and the MIP in Fig. 4 show no mismatch findings. The patient proceeded to RLT with good response to 6 cycles of [^177^Lu]-PSMA-617 (7.4 ± 10% GBq per cycle), with a PSA decline > 50% (pre-therapy 7.7 µg/l, post therapy 1.1 µg/l), with the post-therapy scintigraphy shown also in Fig. 4.

## Discussion

We present a protocol which allows for combined PSMA and FDG-PET/CT imaging within the same session which capitalises on the ability to perform a high-sensitivity and low-dose FDG-PET/CT as part of the same imaging session. The protocol assisted in the identification of one patient who exhibited disseminated bone metastases and a number of liver metastases with low levels of PSMA expression and high FDG avidity, which is consistent with de-differentiated disease and is a relevant consideration in patient selection for PSMA-RLT.

We consider that the implementation of an additional low-dose FDG-PET/CT in the same imaging session makes dual-tracer imaging more feasible, with no need for an additional imaging appointment. A combined low-dose protocol could be more financially viable in centers that do not receive reimbursement for this additional PET/CT examination [6]. Although the lower radiation exposure through lower activities of FDG is of limited value for a patient about to undergo palliative PSMA-RLT, use of lower activities minimises the radiation burden to medical staff and family who must interact with the patient and is consistent with ALARP principles. Moreover, it is our contention that as the providers of a palliative therapy to men in the end-stage of their disease, it is incumbent upon nuclear medicine physicians to draw up treatment plans that minimises  the burdens placed on patients who are approaching the end of a long and exhaustive medical journey. Fundytus et al. argue that the calls placed upon palliative oncology patients’ limited time is underappreciated in the oncological literature [17]. We argue that by combining the additional examination into a single imaging session, not only are we able to better select patients who might benefit from a PSMA-RLT, but can do so in a patient-centered and holistic fashion that places value on their individual comfort and time. We note that our protocol was tolerated well by all patients.

There are a number of options available to the nuclear medicine physician in designing a dual-tracer imaging protocol, and we consider the protocol described herein to be the best compromise between various competing demands while acknowledging its weaknesses. Ideally, both examinations would be performed in a dynamic fashion in order to allow computational extraction of one radiotracers’ uptake compared to another, but we consider the requirement for long-duration dynamic scans to be impracticable for frail patients or in the context of a busy imaging service. An alternative could be to use a low-dose initial PSMA-PET/CT and to inject a higher activity of the additional FDG, analogous to the same-day combined examination performed as part of myocardial perfusion scintigraphy or combined ventilation/perfusion lung scans with Tc-99 m labelled radiopharmaceuticals. However, we consider a standard activity initial PSMA-scan to be of the greatest diagnostic value to the patient and caution that further studies are first required to establish the diagnostic acceptability of low-dose PSMA studies [18, 19]. We counsel against the performance of an initial low-dose FDG PET/CT with additional PSMA-PET/CT, where it would be impossible to differentiate between increasing FDG uptake over time and new foci of PSMA avidity in the second scan [14, 15]. LAFOV systems also offer the possibility to reduce patients’ radiation exposure even further with CT-less attenuation correction [20].

There are some limitations to this protocol. We are cognisant of the difficulty of performing semi-quantitative assessment of FDG avidity where background PSMA activity is present and where prostate cancer lesions exhibit increasing uptake with time [15, 21]. The implications of this on the suitability of pre-therapy PET/CT for dosimetry will have to be addressed by future studies. We take note of the good FDG uptake in non-PSMA-expressing and FDG-avid organs such as the brain as indicators of acceptable bio-distribution and where identification of mismatch lesions with FDG avidity and low PSMA expression is still possible. The high sensitivity of the scanner and ability to scan for 15 min in a single bed position afforded high-quality FDG images despite the lower injected activity. Future studies might address the optimal imaging protocol for theragnostic assessment of PSMA-RLT.

## Conclusion

We introduce a novel imaging protocol which confirms the feasibility of performing single-session dual-tracer imaging with [^68^ Ga]Ga-PSMA-11 and FDG for the assessment of patients undergoing PSMA-RLT.
